# Native kidney BK nephropathy: A case report

**DOI:** 10.1002/ccr3.1982

**Published:** 2019-01-09

**Authors:** Said Al Zein, Hayley Price, Guoli Chen, Gurwant Kaur

**Affiliations:** ^1^ Penn State Health Milton S. Hershey Medical Center Hershey Pennsylvania

**Keywords:** BK virus, bone marrow transplant, immunosuppression, kidney transplant, native BK virus nephropathy

## Abstract

BK virus nephropathy (BKN) is uncommonly reported in native kidneys; mostly reported in bone marrow transplant patients. This case report represents an interesting clinical scenario of biopsy‐proven BKN in native kidneys, in the presence of more than one million copies/mL of BK virus in serum.

## 1 INTRODUCTION

Historically, BK virus nephropathy was thought to be a disease of kidney allografts that only affected renal transplant recipients. BKN is getting more frequently diagnosed as a cause of kidney disease in immunosuppressed patients. Here, we describe a biopsy‐proven native kidney BKN in an allogeneic stem cell transplant patient.

The BK virus was first isolated in 1971 from the urine of a renal transplant patient,^1^ but it was not until early 80s when Polyomavirus (BK and JC viruses) infections were described in renal transplant recipients.^2^, ^3^ At that time, native kidney BKN was considered to occur sporadically with few reports in lymphoma patients and solid organ transplant recipients. More cases of native kidney BKN have been reported in the new millennium with thoughts of under diagnoses of this entity.^4^

In the general population, 70%‐90% of individuals carry anti‐bodies to BK virus.^5^ The primary infection occurs as either a mild respiratory illness or asymptomatic infection during childhood, followed by viral latency usually in the urothelium and renal tubular epithelial cells.^6^, ^7^ Reactivation of BK infection may occur under conditions of immunosuppression, with usual manifestation being hematuria when it is limited to the urothelium.^8^, ^9^ Tissue diagnosis by a kidney biopsy is needed for detection of intrarenal Polyomavirus (PV) using SV40 stain.

## 2 CLINICAL BACKGROUND

Sixty‐seven‐year‐old Caucasian male presented for progressive renal failure and was admitted to oncology floor. His past medical history included mantle cell lymphoma in remission with history of allogeneic hematopoietic stem cell transplant (HSCT) 5 years ago. HSCT is complicated by hypogammaglobulinemia with recurrent upper respiratory viral infections 3 years ago, so he was given monthly infusions of intravenous immunoglobulin G (IVIG) over a period of 6 months. Few months later, he developed chronic, cutaneous graft‐versus‐host disease (GVHD) with manifestation as diffuse scleroderma extending from the lower extremities to the neck. His scleroderma did not improve with outpatient treatment with neither rituximab nor ruxolitinib; however, it did remit and regress to only lower extremity involvement with ibrutinib 280 mg. His clinical course had been complicated with frequent infections including pneumonia which responded to outpatient antibiotics, alongside of a steadily rising creatinine, the ibrutinib was discontinued in interval time.

Medications included mycophenolate mofetil 500 mg twice daily, sirolimus 1 mg every other day, acyclovir 600 mg daily, trimethoprim‐sulfamethoxazole 800‐160 mg twice weekly, and prednisone 10 mg daily.

## 3 CLINICAL COURSE

He was chronically ill appearing. Examination was unremarkable with the exception of skin thickening and mottling in the lower extremities to the knees, consistent with known cutaneous scleroderma.

He presented for progressive renal failure with creatinine level of 4.84 mg/dL (0.7‐1.3 mg/dL), which had steadily risen over the past year from baseline creatinine of 1 mg/dL paralleling progressive fatigue and worsening dyspnea over the previous several months before admission. He had hyperkalemia of 5.7 mmol/L (normal range 3.5‐5 mmol/L) and metabolic acidosis. In the setting of renal failure, his acyclovir, trimethoprim‐sulfamethoxazole, and losartan were held. Urine revealed hematuria (moderate to large hemoglobin with 50+ red blood cells), and only trace proteinuria. Differential diagnosis for his hematuria and acute kidney injury (AKI) included septic/toxic acute tubular necrosis or acute interstitial nephritis, but was considered less likely as his AKI preceded the recent episode of pneumonia and antibiotic treatment. IgA nephropathy was also considered in differential for AKI with hematuria; uncommonly GVHD could also affect kidneys. Additional workup including Hepatitis B and C viral serologies, anti‐nuclear anti‐bodies (ANA < 1:80), C3, and C4 were normal, and antineutrophil cytoplasmic anti‐bodies (ANCA) were undetectable. His blood levels of BK virus yielded over one million copies. BK virus was detected in the urine with PCR showing >390 000 000 copies/mL of the virus DNA. Urine cytology was not done. This was followed by a renal biopsy which confirmed BK virus nephropathy with positive stain for SV 40. His mycophenolate mofetil was discontinued. He was started on leflunomide 100 mg for 3 days and 20 mg daily thereafter (adjusted for renal function). His creatinine slowly but steadily rose throughout his hospital course and was eventually started on hemodialysis (HD). His viral load remained high (BKV serum PCR > 1 000 000 copies/mL). After 2 months, the patient subsequently was started on cidofovir. After 6 weeks of initiating therapy, his viral load started to decline reaching 318 749 copies/mL but he remained HD dependent, became increasingly debilitated and deconditioned on dialysis and eventually passed away 2 weeks after his last clinic visit. The cause of death is unknown; he either passed away at home or at another hospital, likely due to an infection.

## 4 KIDNEY BIOPSY PATHOLOGY

**Figure 1:**
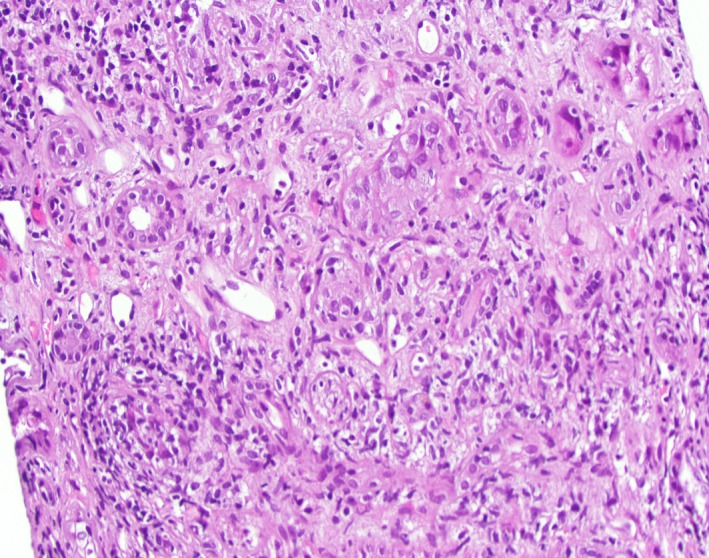
Light microscopy showing marked tubulointerstitial inflammation

**Figure 2:**
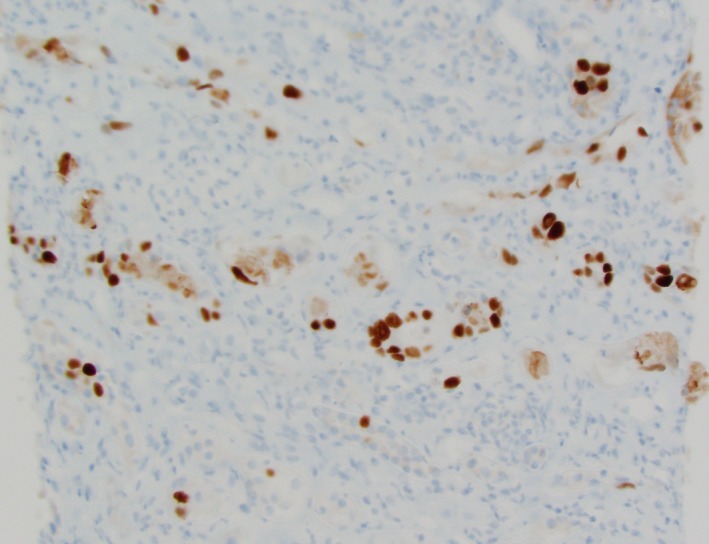
Immunohistochemistry showing positive stain for SV40

Two of 12 glomeruli were globally sclerotic on light microscopy. There was marked tubulointerstitial inflammation (Figure 1) including mainly lymphocytes mixed with plasma cells: Rare neutrophils and eosinophils were noted in a patchy pattern. It showed tubular atrophy and interstitial fibrosis in around 50% of the submitted specimen. Immunohistochemistry was positive for SV40 (Figure 2). Immunofluorescence had no specific staining of IgG, IgA, IgM, C3, kappa, and lambda light chains. Electron microscopy did not reveal any immune deposits.

## 5 DISCUSSION

BK virus nephropathy is uncommonly reported in native kidneys with most of the reported cases occurring in bone marrow transplant patients.^4^ Most of the cases are seen in renal allografts, and most of the literature comes from small series in the transplant population. Diagnosis of BK virus nephropathy involves positive BK virus polymerase chain reaction (PCR) and tissue diagnosis by a kidney biopsy for detection of intrarenal Polyomavirus (PV) replication.^10^ This is done by the detection of T antigen of the Polyomavirus by immunochemistry. T antigen is the only viral protein required for viral replication as all other factors involved in pathogenesis are provided by the infected cells. This immunochemistry detection of T antigen is referred to as SV40‐positive staining, although it is most commonly seen in BK virus (80%) compared to other Polyomaviruses (PV) JC virus (20%) and rarely with simian virus 40 (SV40).^11^ SV40 staining is important to distinguish PV infection from other causes of tubule‐interstitial inflammation like allergic interstitial nephritis.

The usual treatment in transplant patients revolves around decreasing the immune suppression and assessing the response by trending the BK serum viral load which should start to improve within a month or two; full clearance of viremia may take several months. If decreasing immune suppression is not effective, a nonproven therapy, IVIG, is occasionally used^12^ as most IVIG preparations come from BKV exposed donors and contain BKV neutralizing anti‐bodies. Other anti‐viral approaches include the use of leflunomide, a drug that has both immunosuppressive and anti‐viral activity and has shown benefit in few small studies.^13^, ^14^, ^15^ Another alternative is cidofovir, an anti‐viral agent that has been used for HIV‐infected patients with progressive multifocal leukoencephalopathy (PML) resulting from Polyomavirus infection, although it is only Food and Drug Administration approved for treatment of Cytomegalovirus infections. cidofovir has shown benefit in few small studies in the treatment of BKVN^16^, ^17^ and in a retrospective analysis of 18 allo‐HSCT patients with reactivation of BK virus.^18^ Nephrotoxicity is the highest concern when cidofovir is used.

In our case, all immunosuppressive agents were discontinued and the patient was only kept on Leflunomide without much improvement in the serum BK viral load. Then, cidofovir was started and his viral load subsequently showed improving but still high levels. Multiple factors led to deconditioning in our patient including renal failure with dialysis in addition to persistent viremia.

## 6 CONCLUSION

BK Virus nephropathy should be considered as a cause of renal failure in immunocompromised patients who otherwise have no clear diagnosis.

## CONFLICT OF INTEREST

None of the authors’ have any conflicts of interest at the time of publication of this case report.

## AUTHOR CONTRIBUTION

SAZ: contributed to writing of Discussion section. HP: contributed to writing of clinical case and background. GC: contributed to providing pathology slides. GK: contributed to creating the initial case report draft, clinical key message, pathology section, and editing it multiple times.
